# Nutritional Impact and Symptom Profile Associated with 5-Fluorouracil and Capecitabine in Patients with Colorectal Cancer: An Observational Study Using the PG-SGA

**DOI:** 10.3390/diagnostics16213470

**Published:** 2026-10-24

**Authors:** Luis Enrique Sánchez-Diestro, Raquel Macias-Montero, Marta Araujo-Blesa, María Sandra Paniagua-Vivas, Laura Palomares-Nuñez, Jorge Guerrero-Martín

**Affiliations:** 1 Faculty of Medicine and Health Sciences, University of Extremadura, 06071 Badajoz, Spain; raquel.macias@salud-juntaex.es; 2 Department of Biomedical Sciences, Faculty of Medicine and Health Sciences, University of Extremadura, 06071 Badajoz, Spain; 3 Department of Medical Oncology, University Hospital Complex of Badajoz, Extremadura Health Service, 06080 Badajoz, Spain; 4 Grupo Clínico Asociado en Oncología Clínica y Hereditaria, Instituto Universitario de Investigación Biosanitario de Extremadura (INUBE), 06005 Badajoz, Spain; 5 Grupo de Investigación, Docencia e Innovación Biomédica y Sociosanitaria (GIDIBS), Faculty of Medicine and Health Sciences, University of Extremadura, 06071 Badajoz, Spain; maraujob@unex.es; 6 Department of Nursing, Faculty of Medicine and Health Sciences, University of Extremadura, 06071 Badajoz, Spain; 7 Department of Nursing, University Centre of Mérida, University of Extremadura, 06800 Mérida, Spain; mariasanpv@unex.es; 8 Emergency Department of Badajoz University Hospital, University of Extremadura, 06071 Badajoz, Spain; laura.palomares@salud-juntaex.es

**Keywords:** colorectal cancer, 5-fluorouracil, capecitabine, PG-SGA, nutritional status

## Abstract

**Background/Objectives**: Colorectal cancer and its chemotherapeutic treatment may be associated with gastrointestinal toxicity and nutritional deterioration. This study evaluated the association between exposure to 5-fluorouracil (5-FU) or capecitabine and eating-related symptoms and nutritional status. **Methods**: We conducted an observational, cross-sectional study of 91 patients with colorectal cancer receiving active chemotherapy. Clinical, anthropometric, and treatment-related variables were collected. Nutritional status was assessed using the Patient-Generated Subjective Global Assessment (PG-SGA), and body composition was evaluated by multifrequency bioelectrical impedance analysis. Associations between exposure to 5-FU or capecitabine and symptomatic and nutritional variables were analyzed. **Results**: Ninety valid observations were available for the pharmacological analysis. Loss of appetite was more frequent among patients treated with 5-FU than among those not exposed to 5-FU (31.6% vs. 11.5%; *p* = 0.006), as was nausea (26.3% vs. 5.8%; *p* = 0.037). Among patients treated with capecitabine, diarrhea was less frequent than among those not exposed to capecitabine (23.5% vs. 51.3%; *p* = 0.048). **Conclusions**: Exposure to 5-FU and capecitabine was associated with distinct symptom profiles that may be clinically relevant to nutritional status. Multidimensional nutritional assessment using the PG-SGA may facilitate the early identification of nutritional risk during chemotherapy. However, the observational and cross-sectional design precludes causal inference; therefore, these findings require confirmation in prospective, longitudinal studies.

## 1. Introduction

Colorectal cancer (CRC) represents a major global health burden, with more than 2 million new cases and approximately 918,000 deaths reported worldwide [1]. Systemic therapy plays a central role in the management of locally advanced and metastatic disease, with fluoropyrimidines remaining a cornerstone of CRC treatment [2].

5-Fluorouracil (5-FU) and its oral prodrug capecitabine are widely used fluoropyrimidines in CRC and share a common antineoplastic mechanism, although they differ in administration, pharmacokinetics, and toxicity profiles [2,3,4,5]. Clinical trials and meta-analyses have demonstrated broadly comparable oncological efficacy between capecitabine- and 5-FU-based treatments [6,7,8,9,10,11,12]. However, differences in treatment-related toxicity may be clinically relevant from a nutritional perspective, as gastrointestinal and nutrition-impact symptoms such as nausea, diarrhea, anorexia, mucositis, and taste alterations can compromise dietary intake and contribute to nutritional deterioration [13,14].

Nutritional deterioration is common in patients with cancer and may result from the interaction between tumor burden, systemic inflammation, metabolic alterations, reduced dietary intake, and treatment-related adverse effects [13,14,15,16]. In CRC, gastrointestinal symptoms and previous surgery may further compromise nutritional status, potentially affecting treatment tolerance, quality of life, and clinical outcomes [13,15,16]. Consequently, systematic nutritional assessment is recommended as part of comprehensive cancer care [15,16].

The Patient-Generated Subjective Global Assessment (PG-SGA) is a validated multidimensional instrument widely used in oncology that integrates changes in weight and dietary intake, nutrition-impact symptoms, functional capacity, and clinical assessment [17,18,19,20,21]. PG-SGA-defined nutritional deterioration has also been associated with poorer clinical outcomes in patients with cancer [22,23]. Recent studies in patients with CRC receiving chemotherapy have identified substantial nutritional intervention needs and associations between specific chemotherapy regimens, nutrition-impact symptoms, and functional impairment [24,25].

However, while capecitabine and 5-FU have been extensively compared in terms of efficacy and conventional toxicity, their potential differential association with nutritional status and nutrition-impact symptoms assessed using an oncology-specific multidimensional instrument such as the PG-SGA remains insufficiently characterized. This gap is clinically relevant because differences in treatment-related symptoms may affect dietary intake and functional status, dimensions not fully captured by conventional efficacy and toxicity endpoints.

Therefore, the aim of the present study was to compare the nutritional impact associated with 5-FU/LV and capecitabine in patients with CRC using the PG-SGA. Specifically, we evaluated differences in nutritional status and in domains related to dietary intake, nutrition-impact symptoms, and functional capacity to determine whether exposure to these fluoropyrimidines was associated with distinct patterns of nutritional impairment.

## 2. Materials and Methods

### 2.1. Participants and Data Sources

A descriptive, cross-sectional observational study was conducted in adult patients with histologically confirmed stage III or IV colorectal adenocarcinoma who were treated at the Medical Oncology Department of Badajoz University Hospital (Spain). Patient inclusion and data collection were carried out from February to June 2024 during routine clinical follow-up visits.

Eligible participants were adults aged > 18 years with colorectal cancer receiving active chemotherapy, sufficient cognitive capacity to complete the nutritional assessment, and the ability to provide reliable clinical information. All included participants provided written informed consent prior to study enrollment. Patients with severe non-oncological comorbidities that could exert a substantial and independent influence on nutritional status were excluded.

Data were collected through a structured clinical interview and systematic review of electronic medical records. An objective assessment of body composition was also performed using multifrequency bioelectrical impedance analysis. These procedures were integrated into routine clinical care and did not alter the oncological treatment strategy indicated for each patient.

For the present analysis, the study population was stratified according to the fluoropyrimidine administered: 5-fluorouracil plus leucovorin (5-FU/LV) or capecitabine. Patient selection and classification were based on the treatment actually administered during the nutritional assessment period.

### 2.2. Demographic Variables

Sociodemographic variables and baseline clinical characteristics were collected through clinical interviews and review of electronic medical records. Variables included age, sex, body mass index (BMI), tumor anatomical location, and disease stage.

Tumor location was categorized as colon or rectum, whereas disease extent was classified as stage III or IV according to the clinical information available in the medical records.

BMI was calculated from body weight and height recorded at the time of assessment and expressed in kg/m^2^. This variable was included as an anthropometric indicator of nutritional status and as a clinical characteristic potentially associated with the risk of nutritional deterioration.

Demographic and clinical variables were used to characterize the study cohort and assess comparability between groups according to the fluoropyrimidine-based treatment received.

### 2.3. Treatment-Related Variables

Information regarding antineoplastic treatment was obtained from the electronic medical records. The primary exposure variable was the type of fluoropyrimidine administered during the study period, categorized as 5-fluorouracil plus leucovorin (5-FU/LV) or capecitabine.

The concomitant administration of other antineoplastic agents, particularly oxaliplatin and irinotecan, was also recorded when included in the treatment regimen. In the reference study, treatment regimens included combinations based on 5-fluorouracil or capecitabine, together with other agents administered according to the patient’s clinical status and individualized therapeutic strategy.

Therapeutic intent was classified as adjuvant, neoadjuvant, or palliative. Patients were classified according to treatment received. Among patients receiving chemotherapy, two mutually exclusive groups were defined: 5-fluorouracil plus leucovorin (5-FU/LV) and capecitabine. Patients receiving capecitabine did not receive 5-FU/LV, and vice versa. One patient had undergone surgery only and was not receiving chemotherapy; this patient was therefore not included in comparisons between chemotherapy treatment groups.

The presence of symptoms potentially related to treatment toxicity and likely to compromise dietary intake or functional status was also recorded. These included anorexia, nausea, vomiting, taste alterations, diarrhea, constipation, abdominal pain, and asthenia.

Additionally, nutrition-impact symptoms specifically assessed using the Patient-Generated Subjective Global Assessment (PG-SGA) were analyzed, including alterations in food intake, anorexia, constipation, oral mucosal lesions, dysgeusia, intolerance to odors, pain, nausea, vomiting, diarrhea, xerostomia, dysphagia, and early satiety. The Spanish-language version of the PG-SGA (Valoración Global Subjetiva Generada por el Paciente, VGS-GP) was used, following the versions provided by the Spanish Society of Endocrinology and Nutrition (SEEN) and the Spanish Society of Medical Oncology (SEOM).

### 2.4. Nutritional Status Assessment

Nutritional status was assessed using the Patient-Generated Subjective Global Assessment (PG-SGA), a multidimensional instrument specifically developed and validated for nutritional assessment in patients with cancer. The PG-SGA integrates patient-reported information with a clinical assessment performed by healthcare professionals, enabling a comprehensive evaluation of multiple dimensions associated with nutritional deterioration.

The patient-completed component collects information on recent weight changes, alterations in food intake, changes in dietary composition, symptoms potentially affecting food intake, and functional capacity. The healthcare professional component includes clinical assessment of muscle mass and subcutaneous adipose tissue loss, as well as the presence of fluid retention, together with estimation of nutritional requirements and an overall assessment of nutritional status.

The total PG-SGA score was used as the primary variable for characterizing nutritional risk and deterioration. According to the instrument’s established criteria, scores of 0–1 indicated no immediate need for nutritional intervention; scores of 2–3 indicated the need for patient education and specific symptom management; scores of 4–8 indicated the need for specialized nutritional intervention; and scores ≥ 9 indicated a high nutritional risk requiring prioritized intervention.

In addition to the overall score, individual PG-SGA domains related to food intake, nutrition-impact symptoms, and functional capacity were analyzed. Symptoms experienced during the 15 days preceding the assessment that could interfere with adequate oral intake were considered, including anorexia, constipation, oral lesions, taste alterations, intolerance to odors, pain, nausea, vomiting, diarrhea, xerostomia, dysphagia, and early satiety.

Body composition was assessed using multifrequency bioelectrical impedance analysis with the TANITA^®^ RD-545-sv analyzer (TANITA Europe). The following parameters were obtained: fat-free mass, fat mass, skeletal muscle mass, bone mass, and basal metabolic rate. Results were interpreted according to the reference values provided by the manufacturer, adjusted for age and sex, as well as according to criteria established by the European Society for Clinical Nutrition and Metabolism (ESPEN) for the identification of nutritional abnormalities and low muscle mass.

Functional capacity was also considered a specific dimension of nutritional status, consistent with the multidimensional nature of the PG-SGA and its ability to identify functional consequences associated with nutritional deterioration.

Patients were classified according to the chemotherapy regimen received into two mutually exclusive groups: 5-fluorouracil plus leucovorin (5-FU/LV) and capecitabine. Patients receiving capecitabine did not receive 5-FU/LV, and patients receiving 5-FU/LV did not receive capecitabine. Patients who had not yet initiated chemotherapy were not eligible for comparisons between treatment groups.

### 2.5. Statistical Analysis

Statistical analyses were performed using JASP, version 0.17.0.0, implemented in R. Continuous variables were expressed as means and standard deviations or medians and interquartile ranges, depending on their distribution. The normality of continuous variables was assessed using the Kolmogorov–Smirnov test. Categorical variables were described using absolute frequencies and percentages.

A comparative analysis of nutritional status was performed between patients treated with 5-FU/LV and those receiving capecitabine. The primary outcome variable was the total PG-SGA score. Differences between groups in domains related to food intake, nutrition-impact symptoms, and functional capacity were also analyzed.

Categorical variables were compared using Pearson’s chi-square test or Fisher’s exact test, as appropriate based on expected frequencies. For quantitative variables, the corresponding parametric or non-parametric statistical tests were applied according to data distribution. In the original analysis, ANOVA or the Kruskal–Wallis test was used for comparisons of continuous variables.

A two-sided *p* value < 0.05 was considered statistically significant.

### 2.6. Ethical Considerations

The study was conducted in accordance with the ethical principles established by the Declaration of Helsinki and applicable regulations governing biomedical research and the protection of personal data. All participants received detailed information regarding the study objectives and procedures and provided written informed consent prior to enrollment.

The research protocol was reviewed and approved by the Badajoz Health Area of the Extremadura Health Service (Servicio Extremeño de Salud, SES) on 1 February 2024. Subsequently, once constituted, the study was approved by the Badajoz Research Ethics Committee for Medicinal Products (Comité de Ética de la Investigación con Medicamentos de Badajoz) on 21 July 2026, under protocol code CEIm CHUB 005226 OB.

Clinical, anthropometric, and nutritional information was handled confidentially, and appropriate measures were implemented to ensure the protection of participants’ identity and privacy throughout all phases of the study.

## 3. Results

### 3.1. Demographic and Clinical Characteristics of the Study Population

A total of 91 patients with colorectal cancer were included in the overall cohort. Of these, one recently diagnosed patient had not yet initiated chemotherapy, while another patient had undergone surgery only.

**Figure 1:**
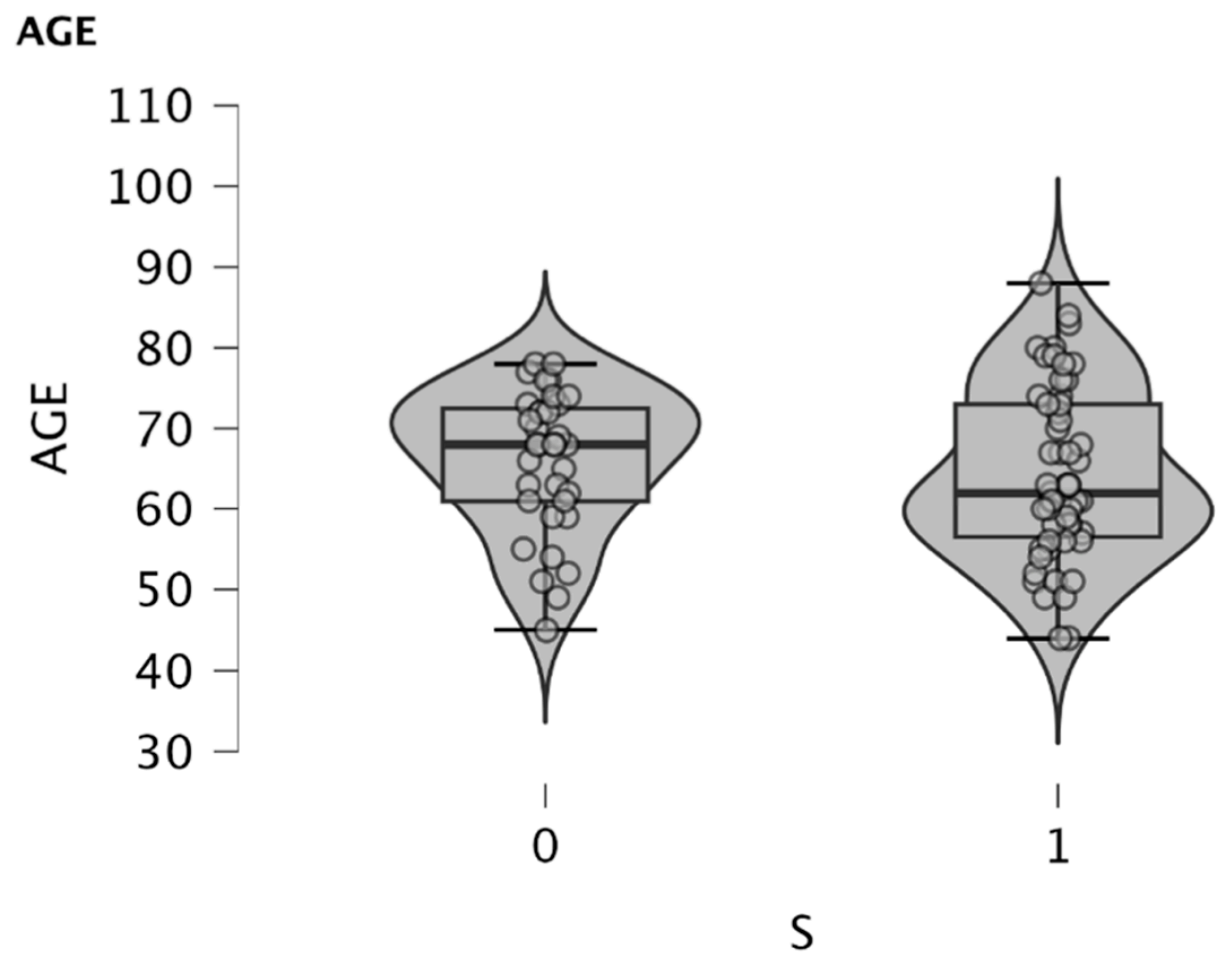
Boxplot of age categorized by sex in the study sample: female (0) and male (1). Source: Authors’ own elaboration.

The mean age of the study population was 64.97 ± 9.92 years, with an interquartile range of 58–73 years. Regarding sex distribution, 56 patients were male (61.54%), and 35 were female (38.46%) (Figure 1/Table 1).

For the analysis of variables related to exposure to 5-fluorouracil and capecitabine, 90 valid observations were available. Exposure to each active agent was analyzed independently. For 5-fluorouracil, category 0 corresponded to patients who were not receiving this treatment, whereas category 1 corresponded to those who were receiving it. The same classification was applied to the corresponding capecitabine variable.

For 5-fluorouracil, 52 patients (57.8%) were classified in category 0 and 38 (42.2%) in category 1. For capecitabine, 39 patients (43.3%) were classified in category 0 and 51 (56.7%) in category 1.

### 3.2. Treatment with 5-Fluorouracil and Capecitabine

Among the 90 patients with available pharmacological data, 38 received 5-FU/LV, and 51 received capecitabine (Table 2). These chemotherapy treatment groups were mutually exclusive. One patient had undergone surgery only and was not receiving chemotherapy; therefore, this patient was not included in comparisons between the two chemotherapy groups. One recently diagnosed patient had not yet initiated chemotherapy and was not eligible for treatment-group comparisons.

The 5-FU/LV and capecitabine treatment groups were mutually exclusive. Patients who were not receiving chemotherapy were excluded from comparisons between chemotherapy treatment groups.

### 3.3. Symptoms According to 5-Fluorouracil Exposure

In the analysis of patients according to 5-fluorouracil exposure, a statistically significant difference was identified in the frequency of food-related problems. This manifestation was present in 36 of 52 patients (69.2%) who were not receiving 5-FU and in 18 of 38 patients (47.4%) who were receiving treatment (χ^2^ = 4.372; *p* = 0.037).

Loss of appetite was reported in 6 of 52 patients (11.5%) without 5-FU treatment and in 12 of 38 patients (31.6%) treated with 5-FU. The comparison between groups showed a statistically significant difference (χ^2^ = 5.511; *p* = 0.019).

**Figure 2:**
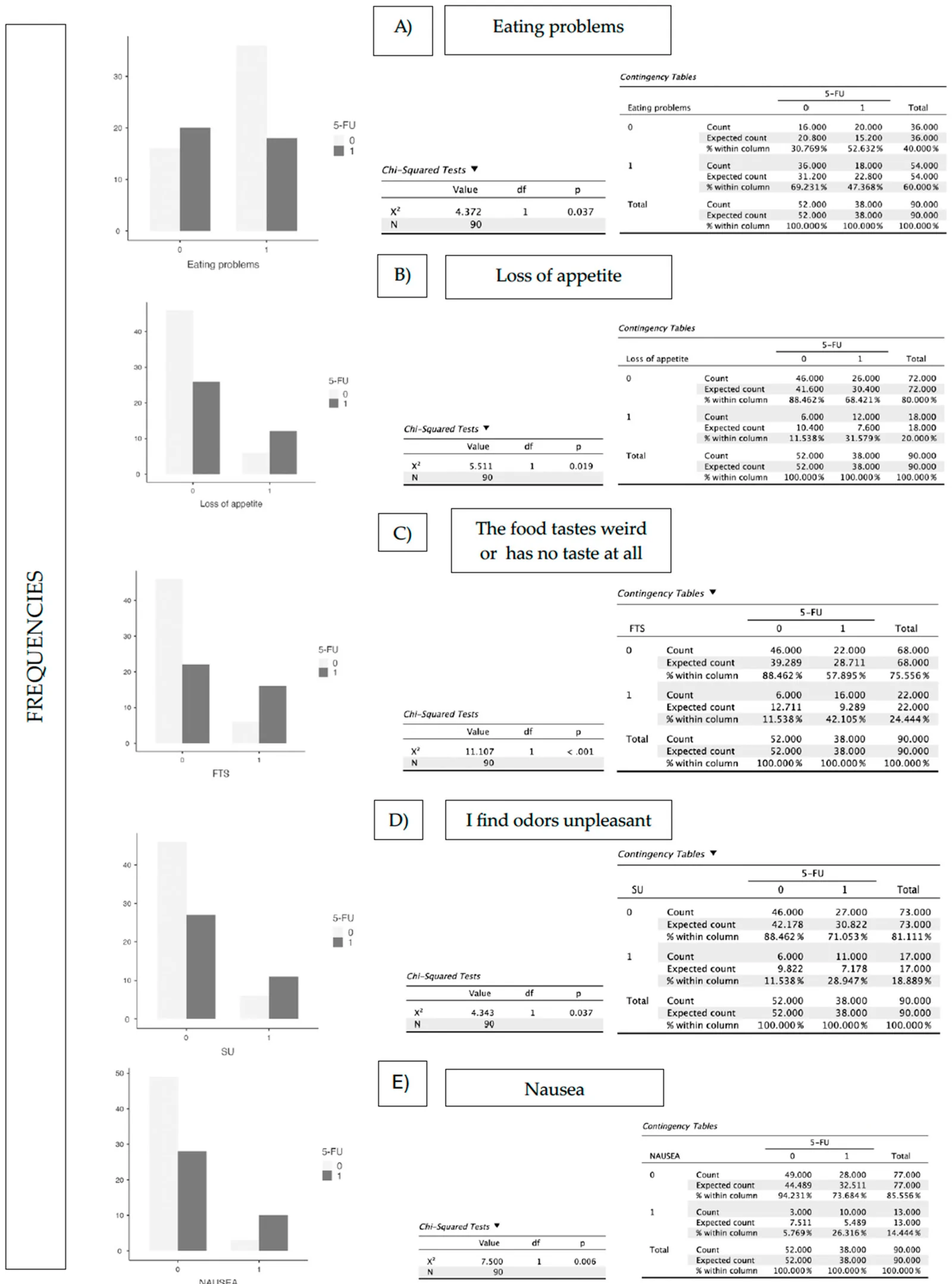
Graphical representation of the statistically significant differences in proportions between patients receiving 5-fluorouracil and those not receiving this treatment with respect to food-related problems, loss of appetite, altered taste (food tasting unusual or having no taste), aversion to odors, and nausea. A value of 0 indicates no administration of 5-fluorouracil, whereas a value of 1 indicates administration. *p*-values obtained using the chi-square (χ^2^) test are indicated. Source: Authors’ own elaboration.

Similarly, a statistically significant difference was observed in the frequency of nausea. This symptom was present in 3 of 52 patients (5.8%) who were not receiving 5-FU and in 10 of 38 patients (26.3%) who were receiving treatment (χ^2^ = 7.500; *p* = 0.006) (Figure 2).

### 3.4. Symptoms According to Capecitabine Exposure

**Figure 3:**
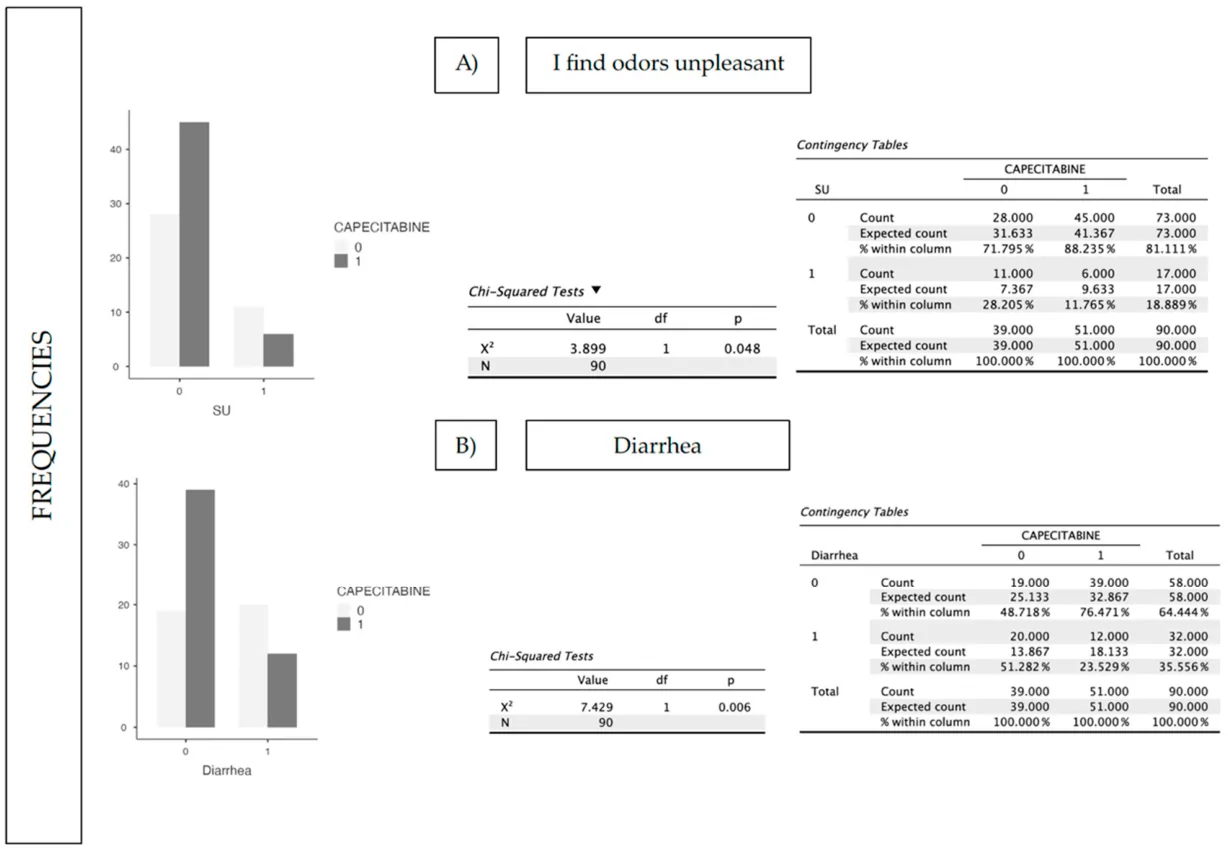
Graphical representation of the statistically significant differences in proportions between patients receiving capecitabine and those not receiving this treatment with respect to the perception of unpleasant odors and diarrhea. A value of 0 indicates no administration of capecitabine, whereas a value of 1 indicates administration. *p*-values obtained using the chi-square (χ^2^) test are indicated. Source: Authors’ own elaboration.

In the analysis of capecitabine exposure, the only variable showing a statistically significant difference was diarrhea. Diarrhea was reported in 20 of 39 patients (51.3%) who were not receiving capecitabine and in 12 of 51 patients (23.5%) who were receiving this treatment (χ^2^ = 7.429; *p* = 0.006) (Figure 3).

The remaining symptom-related variables analyzed showed no statistically significant differences between patients receiving and those not receiving capecitabine, according to the available contingency tables.

### 3.5. Analysis of Treatment-Related Symptoms According to Active Ingredient

The individual analysis of the two active ingredients showed different distributions of the clinical manifestations assessed.

Among patients treated with 5-fluorouracil, feeding-related problems were observed in 47.4%, loss of appetite in 31.6%, and nausea in 26.3%. Among patients who did not receive 5-FU, these manifestations were present in 69.2%, 11.5%, and 5.8%, respectively. The differences were statistically significant for all three variables: feeding-related problems (χ^2^ = 4.372; *p* = 0.037), loss of appetite (χ^2^ = 7.500; *p* = 0.006), and nausea (χ^2^ = 4.343; *p* = 0.037).

Regarding capecitabine, diarrhea occurred in 23.5% of patients receiving the treatment, compared with 51.3% of those who did not receive it (χ^2^ = 3.899; *p* = 0.048) (Table 3).

## 4. Discussion

The present study analyzes the relationship between exposure to 5-fluorouracil (5-FU) or capecitabine and different dimensions related to nutritional status in patients with colorectal cancer (CRC) undergoing chemotherapy. The main finding was the presence of a differential pattern of symptomatology according to the fluoropyrimidine administered.

Among patients exposed to 5-FU, a higher frequency of loss of appetite and nausea was observed, whereas among patients treated with capecitabine, a lower frequency of diarrhea was identified compared with patients who were not exposed. Specifically, loss of appetite was present in 31.6% of patients treated with 5-FU compared with 11.5% of untreated patients (*p* = 0.006), while nausea was observed in 26.3% versus 5.8%, respectively (*p* = 0.037). Regarding capecitabine, diarrhea occurred in 23.5% of exposed patients compared with 51.3% of non-exposed patients (*p* = 0.048). These results should be interpreted within the established evidence showing broadly comparable oncological efficacy between capecitabine and 5-FU/LV, despite differences in their toxicity profiles [7,26].

Therefore, our findings should not be interpreted as evidence of differences in oncological efficacy, but rather as potential differences in symptom burden and nutritional impact.

The results obtained in the present study are particularly interesting when compared with the toxicity data from pivotal trials. In the safety analysis of the two phase III trials, capecitabine was associated with a lower incidence of diarrhea, stomatitis, nausea, alopecia, and severe neutropenia compared with bolus 5-FU/leucovorin [12]. Similarly, the meta-analysis by Petrelli et al., which pooled randomized trials involving patients with advanced CRC, demonstrated equivalent efficacy between capecitabine and 5-FU, with a lower incidence of stomatitis and neutropenia with capecitabine, whereas hand-foot syndrome was significantly more frequent with the oral treatment [27]. These data indicate that the differences between the two fluoropyrimidines are not limited to the route of administration but translate into specific toxicity patterns.

However, our results show a relevant difference compared with these studies. In our cohort, diarrhea was significantly less frequent among patients treated with capecitabine than among those who did not receive this drug (23.5% vs. 51.3%; *p* = 0.048). This finding contrasts particularly with the meta-analysis by Iacovelli et al., which included 23 randomized trials and 15,761 patients and found a higher incidence of severe diarrhea with capecitabine than with 5-FU-based treatments. Among patients with CRC, grade 3–4 diarrhea occurred in 17.0% of patients treated with capecitabine compared with 12.9% of those treated with 5-FU, with a relative risk of 1.46 (95% CI: 1.18–1.81) [28]. This discrepancy is clinically relevant and should be interpreted in light of the methodological differences between these two types of evidence.

This discrepancy should be interpreted in light of the observational and cross-sectional design of our study. Unlike randomized trials, fluoropyrimidine exposure was determined by routine clinical practice, and patients could receive concomitant agents such as oxaliplatin or irinotecan. Therefore, treatment indication and concomitant chemotherapy may have influenced the observed symptom profiles, and the complete treatment regimen should be considered when interpreting nutritional toxicity [29].

In contrast to diarrhea, the findings related to anorexia and nausea observed in our study show a more direct association with variables capable of affecting nutritional status. Patients exposed to 5-FU experienced loss of appetite in 31.6% of cases compared with 11.5% of non-exposed patients and nausea in 26.3% versus 5.8%, respectively. These manifestations are clinically relevant because reduced appetite and nausea may decrease energy and protein intake and, in vulnerable patients, contribute to progressive nutritional deterioration. Nevertheless, it should be noted that the association observed in this study does not allow causality to be determined or establish that 5-FU is directly responsible for these symptoms.

The previous literature is also not entirely consistent in this regard. In the pooled analysis of the two major trials comparing capecitabine with 5-FU/leucovorin, capecitabine was associated with a lower incidence of nausea and diarrhea than the intravenous comparator regimen [12]. Similarly, the pooled analysis of randomized trials conducted by Petrelli et al. did not identify a significant increase in nausea, vomiting, or diarrhea with capecitabine compared with 5-FU [27]. Therefore, the higher frequency of anorexia and nausea associated with 5-FU in our cohort may reflect a specific characteristic of the study population, a consequence of concomitant treatment regimens, or an effect resulting from baseline differences between groups, rather than a reproducible pharmacological difference between the two fluoropyrimidines.

The nutritional interpretation of these findings becomes particularly relevant when considering the role of malnutrition in cancer prognosis. ESPEN recommends systematic nutritional assessment in patients with cancer, with particular attention to reduced intake, weight loss, inflammation, and impaired physical function [14]. In this context, the PG-SGA is a particularly appropriate tool because it integrates information on weight changes, food intake, nutrition-impact symptoms, functional capacity, and clinical assessment of nutritional status [17].

The usefulness of the PG-SGA has been supported by methodological studies and meta-analyses. Bauer et al. initially demonstrated the usefulness of the PG-SGA as a nutritional assessment tool in patients with cancer [17]. Subsequently, a diagnostic meta-analysis comparing different nutritional screening tools reported a pooled sensitivity of 0.964 and specificity of 0.905 for the PG-SGA, which were globally higher than those observed with the other tools analyzed [30]. These findings support the use of the PG-SGA in our study, particularly because the objective was not solely to identify established weight loss but to integrate symptoms, intake, and functionality.

The clinical importance of early identification of nutritional deterioration is supported by evidence linking nutritional and functional impairment with adverse clinical outcomes. A recent meta-analysis found that moderate or high nutritional risk according to the PG-SGA was associated with poorer overall survival and a higher risk of postoperative complications in patients with cancer [23]. Consistently, Karanikki et al. reported that preoperative nutritional assessment provided clinically relevant information regarding postoperative outcomes in patients undergoing surgery for gastrointestinal malignancies [31]. Furthermore, Lidoriki et al. demonstrated that malnutrition and impaired functional status were associated with adverse short-term postoperative outcomes in patients with pancreatic cancer [32]. Although these studies were conducted in surgical populations and are not directly comparable with our chemotherapy-treated cohort, together they reinforce the clinical relevance of integrating nutritional and functional assessment into the management of patients with gastrointestinal cancer. Similarly, de Sousa et al. demonstrated that nutritional indicators were associated with mortality risk in patients with gastric and colorectal cancer [33].

Comparison of our findings with these studies allows us to propose a clinically relevant hypothesis: the choice of fluoropyrimidine may influence not only the classical toxicity recorded in clinical trials but also nutritional dimensions that are less frequently assessed. However, this hypothesis cannot be considered demonstrated by our study. The PG-SGA was designed to identify nutritional risk and deterioration, but an association between a specific drug and certain symptoms is not equivalent to demonstrating that the drug causes malnutrition. This distinction is fundamental, particularly in a cross-sectional study.

Another aspect to consider is that most large comparative trials of capecitabine and 5-FU were primarily designed to evaluate efficacy, survival, and clinical toxicity, whereas our study explicitly incorporates a nutritional perspective. This methodological difference may explain why certain symptomatic variables acquire greater relevance in our study. The PG-SGA allows symptoms, intake, and functionality to be considered jointly, dimensions that are not necessarily captured by traditional toxicity criteria used in clinical trials [17,30].

From a clinical perspective, these findings support an integrated nutritional assessment strategy during chemotherapy. The identification of anorexia, nausea, diarrhea, or other symptoms with a potential impact on food intake should trigger systematic nutritional assessment, particularly in patients with advanced disease, previous weight loss, or functional impairment. The ESPEN recommendations specifically identify early detection of nutritional risk and management of symptoms that compromise food intake as central components of nutritional support in oncology [14].

Nevertheless, the limitations of the study should be carefully considered. The sample size was small, with 91 patients included and 90 valid observations for the exposure analyses of 5-FU and capecitabine. In addition, the cross-sectional design prevents the establishment of a temporal relationship between chemotherapy administration and the onset of symptoms. The lack of randomization introduces potential selection bias and confounding by indication, while concomitant administration of oxaliplatin, irinotecan, or other treatments makes it difficult to attribute symptoms to a single molecule. Furthermore, the potential influence of concomitant chemotherapy cannot be completely excluded, and individual stratification between stage III and IV disease was not available for multivariable adjustment; therefore, the observed associations should not be interpreted as independent causal effects of fluoropyrimidine exposure. Finally, no longitudinal assessment was available to determine whether symptomatic alterations subsequently translated into weight loss, loss of muscle mass, treatment interruption, or deterioration in quality of life.

Therefore, future studies should adopt a prospective, longitudinal design, incorporating a baseline nutritional assessment before treatment initiation and serial assessments throughout chemotherapy cycles. It would also be advisable to analyze the complete treatment regimen, adjust for disease stage, treatment intent, line of treatment, tumor location, age, and functional status, and complement the PG-SGA with objective measures of muscle mass, such as body composition analysis using computed tomography, as well as handgrip strength and physical performance tests.

In conclusion, our results suggest that exposure to 5-FU and capecitabine may be associated with different symptom patterns of potential nutritional relevance. The higher frequency of anorexia and nausea observed with 5-FU contrasts with the lower frequency of diarrhea observed with capecitabine in our cohort. However, the latter finding differs from the results of large trials and meta-analyses that have reported a higher risk of severe diarrhea with capecitabine [28] and therefore should not be interpreted as a protective effect of the drug. Overall, the data support the need to incorporate multidimensional nutritional assessment into the monitoring of fluoropyrimidine toxicity, but these findings should be considered hypothesis-generating until confirmed by larger prospective studies with adequate control of confounding factors.

## 5. Conclusions

In patients with colorectal cancer undergoing chemotherapy, exposure to 5-fluorouracil and capecitabine was associated with different symptom profiles of potential nutritional relevance, with a higher frequency of anorexia and nausea observed among patients treated with 5-fluorouracil and a lower frequency of diarrhea among those exposed to capecitabine. These findings suggest that gastrointestinal toxicity associated with fluoropyrimidines may have implications for food intake and the risk of nutritional deterioration; therefore, multidimensional nutritional assessment using the PG-SGA may contribute to early detection and comprehensive management. However, given the observational and cross-sectional nature of the study, these associations do not establish causality. Larger prospective and longitudinal studies are needed to confirm these findings and determine their impact on treatment tolerance, functional status, and oncological outcomes.

## Figures and Tables

**Table 1:** Demographic characteristics of the study population. Demographic data were obtained from the overall cohort of the original study.

Characteristic	Total Population
Patients, *n*	91
Age, mean ± SD, years	64.97 ± 9.92
Age, P25-P75, years	58–73
Men, n (%)	56 (61.54)
Female, n (%)	35 (38.46)

**Table 2:** Exposure to 5-fluorouracil and capecitabine. The analyses correspond to 90 valid observations.

Treatment	Does not Receive Treatment, n (%)	Receives Treatment, n (%)
5-Fluorouracil	52 (57.8)	38 (42.2)
Capecitabine	39 (43.3)	51 (56.7)

**Table 3:** Symptomatic variables significantly associated with 5-fluorouracil or capecitabine. Note: The results are presented exclusively in descriptive terms. No causality is established, nor is the clinical direction of the associations interpreted in this section.

Active Ingredient	Variable	Without Treatment, n (%)	With Treatment	χ^2^	*p*
5-FU	Feeding-related problems	36/52 (69.2)	18/38 (47.4)	4.372	0.037
5-FU	Loss of appetite	6/52 (11.5)	12/38 (31.6)	7.500	0.006
5-FU	Nausea	3/52 (5.8)	10/38 (26.3)	4.343	0.037
Capecitabine	Diarrhea	20/39 (51.3)	12/51 (23.5)	3.899	0.048

## Data Availability

The original contributions presented in this study are included in the article. Further inquiries can be directed to the corresponding author.

## Abbreviations

The following abbreviations are used in this manuscript:

5-FU	5-Fluorouracil
CRC	Colorectal Cancer
GLOBOCAN	Global Cancer Observatory
LV	Leucovorin
ESPEN	European Society for Clinical Nutrition and Metabolism
ASCO	American Society of Clinical Oncology
PG-SGA	Patient-Generated Subjective Global Assessment
GLIM	Global Leadership Initiative on Malnutrition
BMI	Body Mass Index
